# Indigenous food systems of the Garo Hills of Meghalaya: nutritional composition and potential health benefits of traditional green leafy vegetables

**DOI:** 10.3389/fnut.2026.1829858

**Published:** 2026-09-03

**Authors:** Sunanda Nongthombam, Natasha R. Marak, Chingakham Basanti Devi, Chenxiang Rimchi N. Marak, Pavana Kumar, Namita Singh, Puspita Das

**Affiliations:** 1 Central Agricultural University, Imphal, India; 2 North East Society for Agroecology Support (NESFAS), Shillong, India

**Keywords:** agrobiodiversity, dietary diversity, green leafy vegetables, indigenous food systems, neglected and underutilized species, Northeast India

## Abstract

Indigenous food systems play a vital role in sustaining dietary diversity and nutritional security among tribal populations, yet their contributions remain inadequately documented. This study aimed to evaluate the nutritional composition and potential health benefits of traditional green leafy vegetables (GLVs) within the indigenous food system of the Garo Hills, Meghalaya. Participatory rural appraisal techniques, including focus group discussions and four-cell analysis, were employed in two tribal villages to identify culturally significant GLVs and document associated traditional knowledge. A total of 20 GLVs were recorded, of which 10 were selected for detailed nutrient and phytochemical analysis using standard methods. The results revealed significant variation (*p* ≤ 0.05) in nutrient composition among species, with several GLVs exhibiting high levels of protein, iron, calcium, and dietary fiber. Additionally, substantial concentrations of phenolics, flavonoids, and vitamin C indicate strong antioxidant potential. These properties suggest a role in reducing oxidative stress and addressing micronutrient deficiencies linked to conditions such as anemia, diabetes, and hypertension. The findings highlight the dual nutritional and functional significance of indigenous GLVs and underscore the importance of integrating traditional knowledge with scientific validation. Strengthening indigenous food systems through the promotion of these vegetables may contribute to improved nutrition security, health outcomes, and sustainable food systems.

## 1 Introduction

### 1.1 Benefits and significance of GLVs

Green leafy vegetables (GLVs) represent one of the most important categories of crops that offer diverse benefits to local communities and agricultural systems. They are widely consumed in traditional diets and medicinal practices and play a critical role in ensuring food and nutritional security, enhancing dietary diversity, supporting rural livelihoods, and sustaining cultural traditions. GLVs are rich sources of dietary fiber, essential micronutrients such as vitamins and minerals, and bioactive compounds including phytochemicals with antioxidant and anti-inflammatory properties. Furthermore, many GLVs are well adapted to marginal environments, require minimal external inputs, and exhibit resilience to climatic stresses, making them important components of sustainable agricultural systems. These attributes collectively contribute to improved nutritional status, better health outcomes, and enhanced resilience of food systems (1, 2).

With these nutritional and ecological advantages, GLVs have increasingly attracted both local and global attention for their promotion. International initiatives such as the Future Smart Food program led by the Food and Agriculture Organization emphasize the importance of rediscovering neglected and underutilized crops to address hunger, malnutrition, and environmental sustainability (3–5).

Despite these advantages, many traditional GLVs remain underutilized and under-researched. They are often categorized under neglected and underutilized species (NUS), a group of crops that have received limited attention from scientific research, agricultural development programs, and policy frameworks. However, within this broader category, GLVs represent a nutritionally significant subgroup that is closely linked to indigenous knowledge systems and local food cultures.

### 1.2 Context: GLVs in Meghalaya

The state of Meghalaya in northeastern India is recognized for its rich biodiversity and diverse indigenous food systems, including a wide variety of wild and semi-cultivated GLVs traditionally used by local communities, particularly the Garo tribe. These vegetables are commonly gathered from forests, cultivated in shifting agricultural systems, or grown in home gardens. Many of these species contribute significantly to household food security and dietary diversity in rural communities (6, 7).

### 1.3 Problem statement

Despite their numerous benefits, many GLVs remain poorly documented and are gradually declining in use due to changing food habits, agricultural transitions, and increasing reliance on market-oriented crops. Traditional knowledge related to their identification, preparation, and medicinal use is primarily preserved among older community members, particularly women and experienced foragers, and is at risk of erosion. Moreover, these crops continue to be neglected and underutilized, receiving limited attention from scientific research, agricultural development programs, and policy frameworks. Scientific evidence linking indigenous knowledge with nutritional and phytochemical composition also remains limited for many traditional vegetables of the Garo Hills. This gap highlights the need for integrated studies that combine ethnobotanical knowledge with scientific nutritional validation.

Despite their numerous benefits, many GLVs remain poorly documented and are gradually declining in use due to changing food habits, agricultural transitions, and increasing reliance on market-oriented crops. Traditional knowledge related to their identification, preparation, and medicinal use is primarily preserved among older community members, particularly women and experienced foragers, and is at risk of erosion. Moreover, these crops continue to be neglected and underutilized, receiving limited attention from scientific research, agricultural development programs, and policy frameworks. Scientific evidence linking indigenous knowledge with nutritional and phytochemical composition also remains limited for many traditional vegetables of the Garo Hills. While this gap highlights the need for integrated studies that combine ethnobotanical knowledge with scientific validation, such efforts must be undertaken with due recognition of Indigenous communities as knowledge custodians and in alignment with ethical frameworks governing access and use of traditional knowledge, including the Convention on Biological Diversity and the Nagoya Protocol (8, 9).

### 1.4 Objectives

Against this backdrop, the objectives of the present study were threefold:

1. To identify and prioritize neglected and underutilized green leafy vegetables through participatory community-based approaches.
2. To document indigenous traditional knowledge related to their culinary and medicinal uses.
3. To evaluate the nutrient and phytochemical composition of selected species in order to assess their potential contribution to dietary diversity and nutrition security.

## 2 Materials and methods

### 2.1 Study area

The study was conducted in the West Garo Hills and East Garo Hills districts of Meghalaya, India. Both districts fall within the Nokrek Biosphere Reserve, a UNESCO-designated Biosphere Reserve under the Man and the Biosphere (MAB) Program, and lie within the broader Eastern Himalayan biodiversity hotspot. The region experiences a humid subtropical to tropical monsoon climate, with a mean annual rainfall ranging from approximately 2,500 to 3,800 mm and temperatures generally varying between 10 °C and 35 °C, depending on season and elevation. The natural vegetation comprises predominantly tropical evergreen, semi-evergreen, tropical moist deciduous, and subtropical broad-leaved forests, supporting a rich diversity of native flora and wild edible plant species.

Agriculture in the region is predominantly rain-fed and is characterized by traditional shifting cultivation (*jhum*), home gardens, and smallholder mixed farming systems. These agroecosystems, together with adjacent forests and fallow lands, support a rich diversity of cultivated, semi-wild, and wild edible plant species that contribute significantly to local food and nutrition security. The region is predominantly inhabited by the matrilineal Garo tribe, whose livelihoods are closely linked to traditional agriculture, forest resources, and Indigenous food systems.

**Figure 1:**
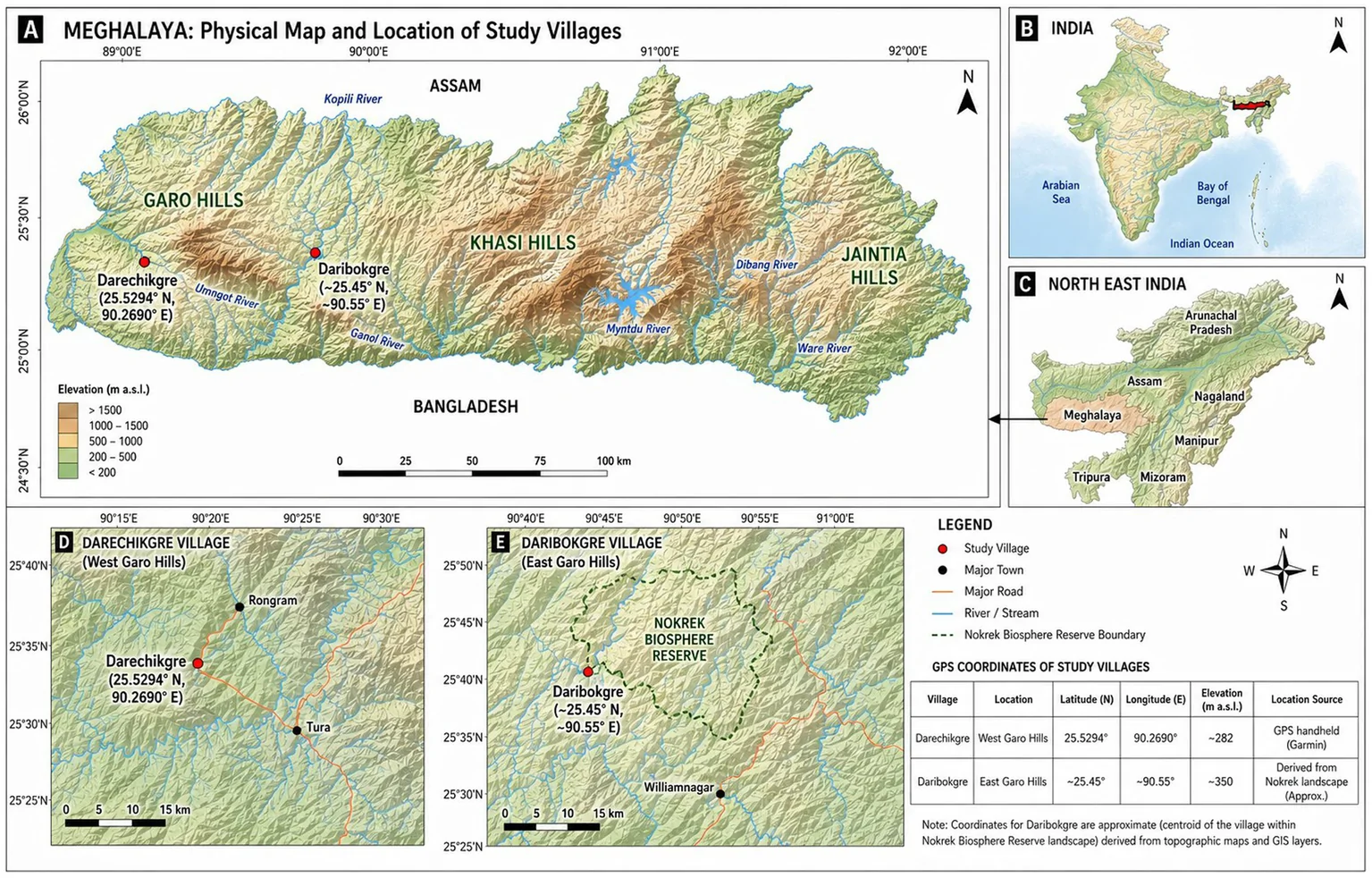
Physical map of Meghalaya showing the location of the study villages, Darechikgre and Darobokgre, India. Source: Cartographic map prepared by the author based on field location information and GIS/topographic data.

A total of two villages were purposively selected for the study (Figure 1):

- Darechikgre village in West Garo Hills (25.5294° N, 90.2690° E).
- Daribokgre village in East Garo Hills (approximately 25.45° N, 90.55° E).

The villages were selected based on (i) the continued practice of traditional *jhum* cultivation, (ii) the presence of knowledgeable community members possessing Indigenous Traditional Knowledge (ITK) related to green leafy vegetables, (iii) the continued use of indigenous edible plant species in local diets, and (iv) the willingness of community members to participate in the study.

### 2.2 Study design

An exploratory research design combining participatory approaches and laboratory-based nutritional analysis was employed. Participatory rural appraisal methods were used to identify and prioritize neglected green leafy vegetables, while experimental laboratory analysis was conducted to evaluate their nutrient composition.

### 2.3 Participatory identification of green leafy vegetables

One Focus Group Discussion (FGD) was conducted in each of the two study villages, resulting in a total of two FGDs. Participants were purposively identified with the assistance of local village leaders and community facilitators and included experienced farmers, foragers, village elders, and women recognized for their extensive knowledge of Indigenous Traditional Knowledge (ITK) related to food plants. The FGD in Darechikgre comprised 15 participants (9 males and 6 females), while the FGD in Daribokgre comprised 7 participants, all of whom were women, giving a total of 22 participants.

During each FGD, participants prepared an inventory of cultivated, semi-wild, and wild edible plant species known within their communities. In Daribokgre, participants listed 41 crop species from shifting cultivation (*a’ba*) and 33 species collected from forests (*burung*). In Darechikgre, 47 crop species from shifting cultivation and 62 forest species were documented. Through facilitated discussion and community consensus, neglected and underutilized green leafy vegetables (GLVs) were identified and prioritized based on their frequency of consumption, cultural significance, perceived nutritional and medicinal value, and continued use within the community.

A shortlist of 15 neglected and underutilized GLVs was generated in each village. Consolidation of the two village lists resulted in the documentation of 20 unique neglected and underutilized GLVs, of which 10 species were common to both villages, while the remaining species were unique to one of the two study sites.

### 2.4 Four-cell analysis

Four-Cell Analysis (FCA) was used as a participatory tool to assess the distribution and cultivation prevalence of the identified green leafy vegetables (GLVs) within the study villages. The analysis was based on two criteria:

1. Relative area under cultivation.
2. Relative number of households cultivating the species.

Based on these parameters, the GLVs were grouped into four categories:

- Large area, many households.
- Large area, few households.
- Small area, many households.
- Small area, few households.

Classification into the categories “many households” and “few households” was based on community perceptions regarding the relative number of households cultivating a particular species, while “large area” and “small area” referred to the relative extent of cultivation as identified during focus group discussions. No numerical scores or weights were assigned to the criteria; rather, classification was achieved through consensus among participants. Both criteria (relative area under cultivation and relative number of households cultivating the species) were considered with equal importance during the participatory classification. The FCA focused on cultivated GLVs occurring within home gardens and *jhum*-based agricultural systems and did not include exclusively foraged forest species.

The analysis provided a relative community-level assessment of cultivation patterns and helped identify species that may be more vulnerable to decline due to limited cultivation and restricted household-level adoption.

### 2.5 Documentation of indigenous traditional knowledge

Prior informed consent was obtained from all participants before interviews and focus group discussions. Participants were informed of the objectives of the study, and participation was voluntary. Indigenous Traditional Knowledge was documented with community permission and is presented with due recognition of community ownership and knowledge custodianship. The study adhered to ethical principles for research involving human participants.

### 2.6 Agrobiodiversity walk

One agrobiodiversity walk and one focus group discussion (FGD) were conducted in each village with the participation of village elders, farmers, and local knowledge holders. The agrobiodiversity walks enabled the verification of species identified during the FGDs and facilitated the identification of additional species occurring in forests, home gardens, and shifting cultivation (jhum) areas.

### 2.7 Nutrient profiling of selected GLVs

Ten GLVs were selected for detailed nutritional analysis based on their availability, cultural significance, and frequency of consumption.

A total of 20 GLVs were documented through field surveys and participatory methods. Of these, 10 species were selected for detailed nutritional analysis based on predefined criteria, including frequency of citation among respondents, cultural significance (culinary and medicinal uses), and availability during the study period for fresh sample collection. The selected species included both commonly reported GLVs across the two study villages as well as a subset of location-specific species to capture local variation. Species that were infrequently cited, seasonally unavailable, or could not be collected in sufficient quantities were excluded from laboratory analysis. Among the selected species, species such as *Bauhinia acuminate* and *Persicaria chinensis* were common to both villages, while species such as *Fagopyrum esculentum* and *Manihot esculenta* were unique to individual sites. These criteria ensured that laboratory resources were focused on culturally important species while adequately representing the diversity documented during field investigations.

#### 2.7.1 Sample preparation

Plant samples were taxonomically identified in consultation with experts from the North East Society for Agroecology Support (NESFAS), ensuring accuracy in species identification. The collected samples were washed with distilled water, shade-dried to remove surface moisture, and subsequently oven-dried at 50–65 °C until constant weight was achieved. The dried samples were ground into a fine powder using a commercial grinder (Bajaj GX-1, 500 W Mixer Grinder). The powdered samples were then stored in airtight containers in a desiccator for a period of 7 days prior to analysis to prevent moisture absorption and maintain sample stability.

### 2.8 Proximate analysis

Proximate analysis refers to the determination of the major nutritional components of food, including moisture, ash, crude protein, crude fat, crude fiber, and carbohydrates. In the present study, proximate composition of the selected green leafy vegetables was determined using standard methods of the Association of Official Analytical Chemists (10).

Moisture content was determined using a hot air oven (AOAC 925.10) by drying samples at 105 °C until constant weight was achieved. Ash content was estimated using a muffle furnace (AOAC 923.03) at 550 °C. Crude protein was determined by the Kjeldahl method (AOAC 2001.11/984.13) using a KelPlus Kjeldahl digestion and distillation system (Make: Pelican Equipments, India; Model: KEL PLUS Model: KES 06LE). Crude fat was analyzed using a Soxhlet extraction apparatus (AOAC 920.39) (Make: Pelican Equipments, India; Model: SOCSPLUS SCS 06RB) with appropriate solvent. Crude fiber was determined using a fiber estimation apparatus (AOAC 962.09) (Make: Pelican Equipments, India; Model: KEL PLUS Model: FIBRA PLUS FFS 08) following sequential acid and alkali digestion. Carbohydrate content was calculated by difference, and energy value was estimated using Atwater factors.

### 2.9 Mineral analysis

Calcium (Ca) and iron (Fe) contents were determined using Atomic Absorption Spectrophotometer (AAS; Model AA-7800) following AOAC (2000) standard procedures. Approximately 1 g of dried powdered sample was subjected to wet digestion using a mixture of concentrated nitric acid (HNO₃) and perchloric acid (HClO₄) until a clear solution was obtained. The digested samples were filtered and diluted to a known volume with distilled water prior to analysis. Calcium and iron concentrations were quantified at wavelengths of 422.7 nm and 248.3 nm, respectively, using calibration curves prepared from standard solutions of each mineral. Results were expressed as mg/100 g dry weight (DW). All analyses were performed in triplicate, and the mean values were used for statistical analysis.

### 2.10 Determination of bioactive compounds

The bioactive compounds of the selected green leafy vegetables were evaluated by estimating vitamin C, total phenolic content (TPC), and total flavonoid content (TFC) using standard analytical methods.

Vitamin C was determined according to AOAC Official Method 967.21 using 2,6-dichlorophenol-indophenol (DCPIP) titration. Samples were extracted using metaphosphoric acid (or a metaphosphoric acid–acetic acid mixture) to stabilize ascorbic acid prior to analysis. Standard ascorbic acid solutions were used for calibration.

Total phenolic content (TPC) was determined using the Folin–Ciocalteu method. Approximately 0.5 g of sample was extracted with methanol, reacted with Folin–Ciocalteu reagent followed by sodium carbonate, and absorbance was measured at 765 nm using a UV–Vis spectrophotometer. Results were expressed as mg gallic acid equivalents (GAE)/100 g.

Total flavonoid content (TFC) was determined using the aluminum chloride colorimetric method. Approximately 0.5 g of sample was extracted with methanol and reacted with aluminum chloride solution. Absorbance was measured at 415 nm using a UV–Vis spectrophotometer, and results were expressed as mg quercetin equivalents (QE)/100 g.

### 2.11 Statistical analysis

All laboratory analyses were conducted in triplicate, and the mean values obtained from the three replicates were used for statistical analysis. Data were analyzed using IBM SPSS version 20.

Descriptive statistics including mean and standard deviation were calculated. One-way Analysis of Variance (ANOVA) was performed to determine significant differences among samples. Duncan’s Multiple Range Test was applied to compare treatment means at a significance level of *p* ≤ 0.05.

## 3 Results

### 3.1 Identification of green leafy vegetables

Table 1 presents the identification of neglected and underutilized GLVs across the study villages, revealing variation in species occurrence, sources, and utilization patterns. A total of 20 species were documented, of which 10 were common to both villages, indicating both shared and location-specific plant use patterns.

**Table 1:** Garo name, common name, scientific name, and family of the identified 20 NUGLVs.

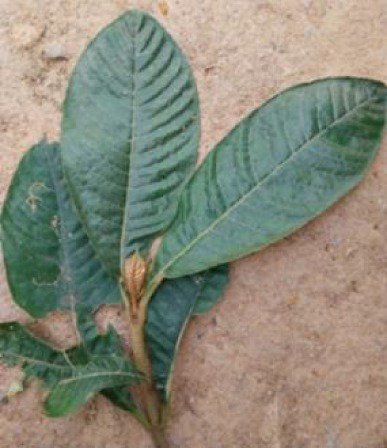 Garo name: *Me·bitchi* Common name: Elliptic rhynocotecum Scientific name: *Rhynchotechumellipticum* Family: Gesneriaceae	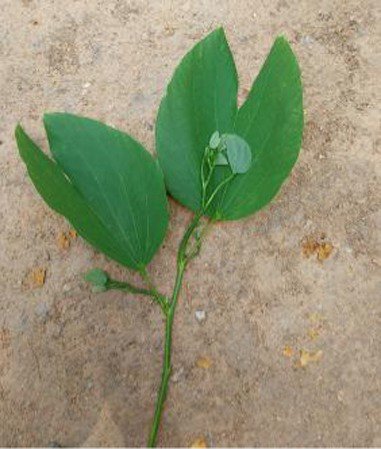 Garo name: *Me·gong Bijak* Common name: Dwarf White Orchid Tree/butterfly tree Scientific name: *Bauhinia acuminata* Family: Fabaceae
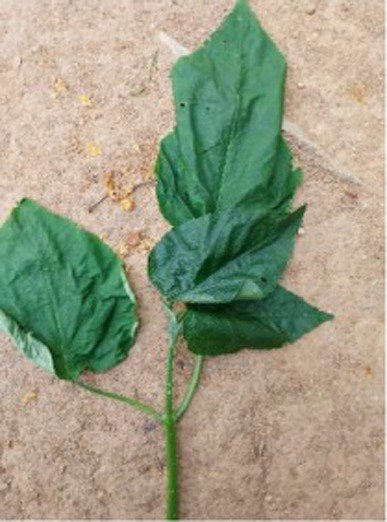 Garo name: *Donggam* Common name: East Indian glory bower Scientific name: *Clerodendrum indicum* Family: Lamiaceae	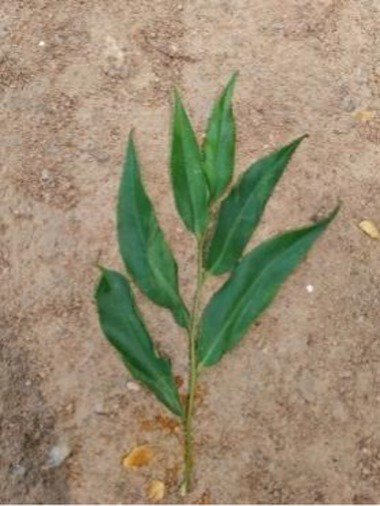 Garo name: *Derimet/Dadarimit*
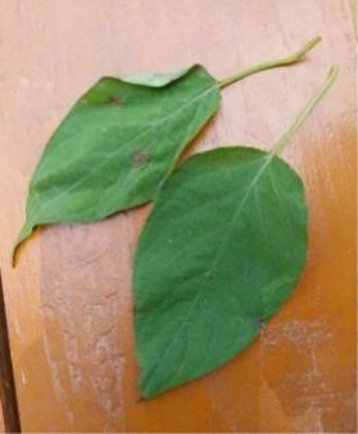 Garo name: *Pasim* Common name: Skunk Vine/Stink Vine Scientific name: *Paederiafoetida* Family: Rubiaceae	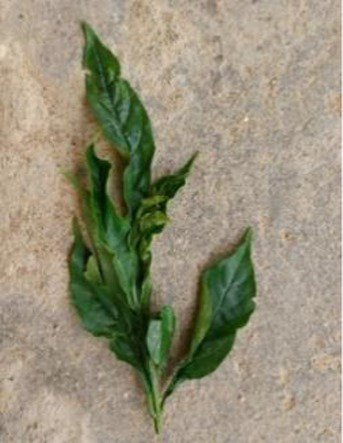 Garo name: *Balmatchi/Balmitchi* Common name: Anamu Scientific name: *Petiveriaalliacea* Family: Petiveraceae
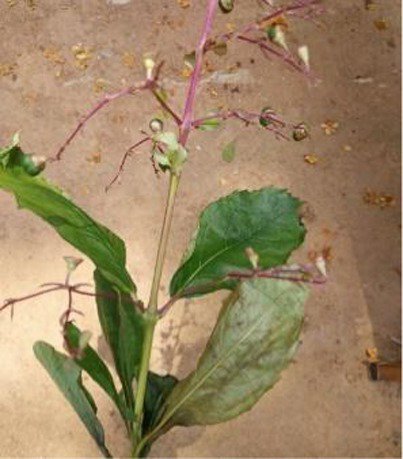 Garo name: *Agunjareng/Aginjaleng* Common name: Blue fountain bush Scientific name: *Rotheca serrata* Family: Lamiaceae	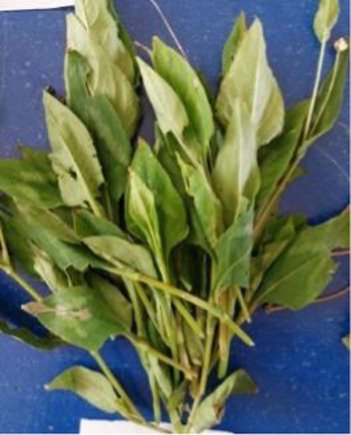 Garo name: *Me·kridunok/Me·kridonok* Common name: Creeping smartweed/Chinese knotweed Scientific name: *Persicaria chinensis* Family: Polygonaceae
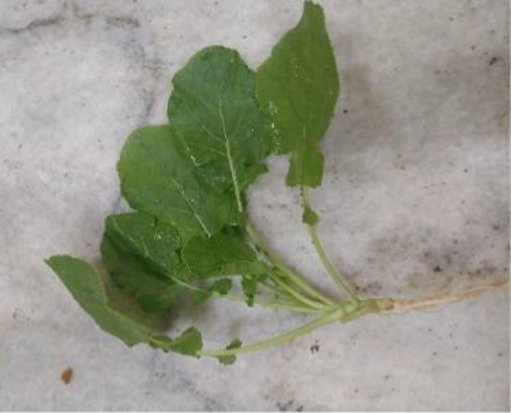 Garo name: *Me·jak* Common name: Bitter Dock Scientific name: *Rumex obtusifolius* Family: Polygonaceae	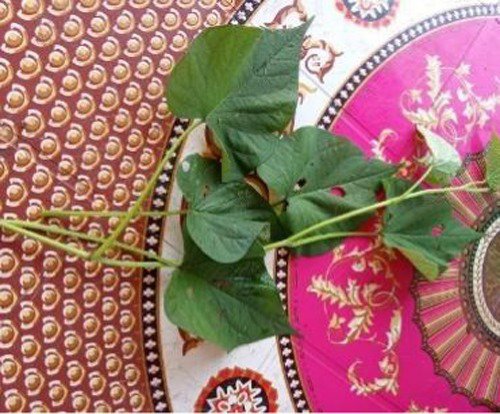 Garo name: *Ta·milangbijak* Common name: Sweet potato leaves Scientific name: *Ipomoea batatas* Family: Convolvulaceae
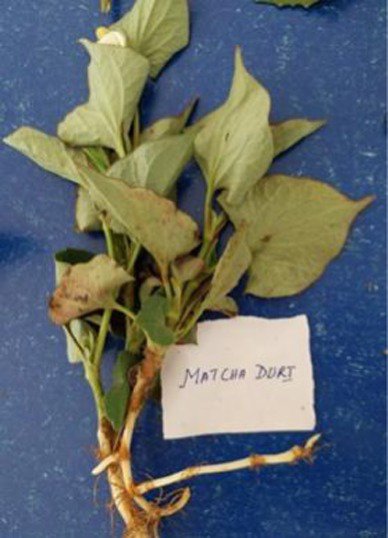 Garo name: *Matchaduri/Chadli* Common name: Fish Mint Leaves/Chameleon Plant Scientific name: *Houttuynia cordata* Family: Saururaceae	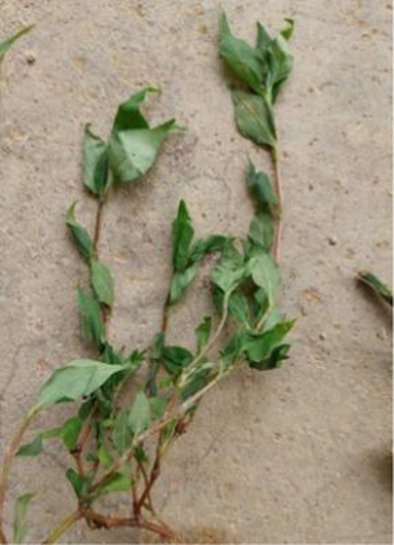 Garo name: *Samjalik* Common name: False mellow Scientific name: *Malvastrumtricuspidatum* Family: Malvaceae
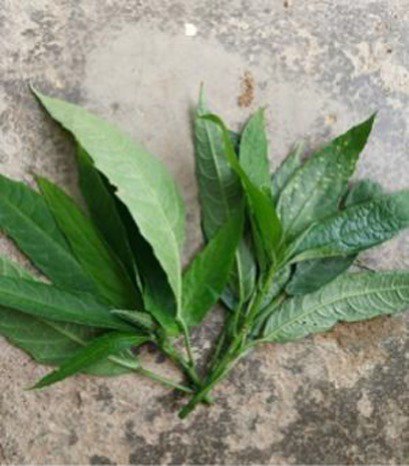 Garo name: *Medongde* Common name: Wallich’s glory bower Scientific name: *Clerodendrum wallichii* Family: Lamiaceae	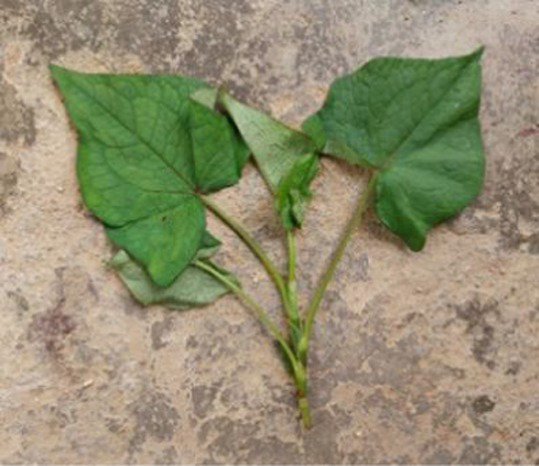 Garo name: *Samdambong* Common name: Buckwheat leaves Scientific name: *Fagopyrum esculentum* Family: Polygonaceae
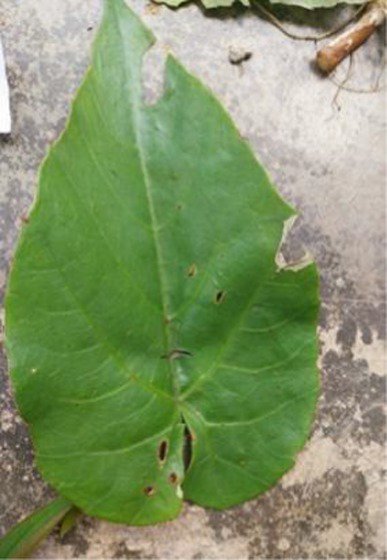 Garo name: *Derikep*	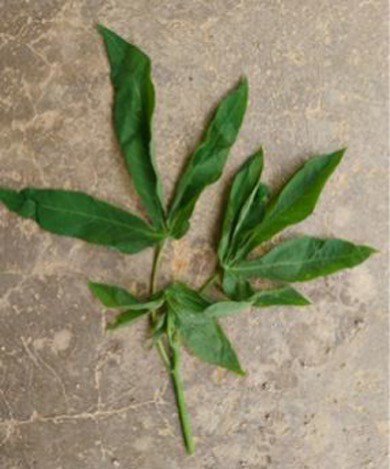 Garo name: *Ta·bolchubijak* Common name: Tapioca leaves Scientific name: *Manihot esculenta* Family: Euphorbiaceae
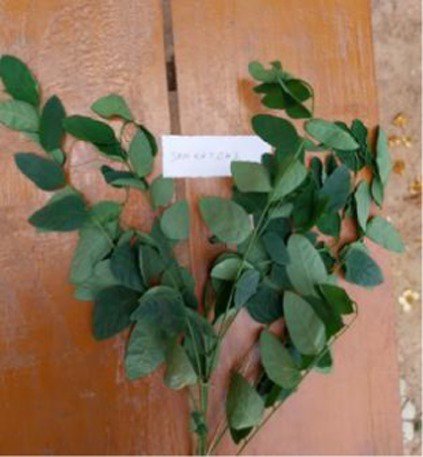 Garo name: *Samkatchi* Common name: Sweet leaf Scientific name: *Sauropusandrogynus* Family: Phyllanthaceae	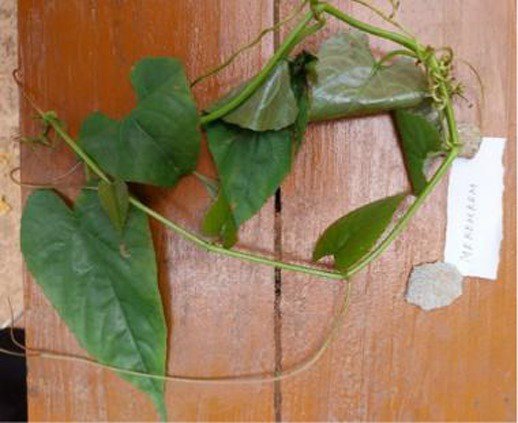 Garo name: *Me·kemkem* Common name: East Himalayan Begonia Scientific name: *Begonia roxburghii* Family: Begoniaceae
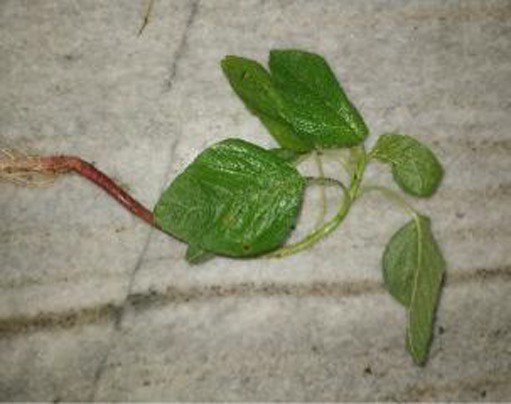 Garo name: *Denggasak* Common name: Amaranth Scientific name: *Amaranthus viridis* Family: Amaranthaceae	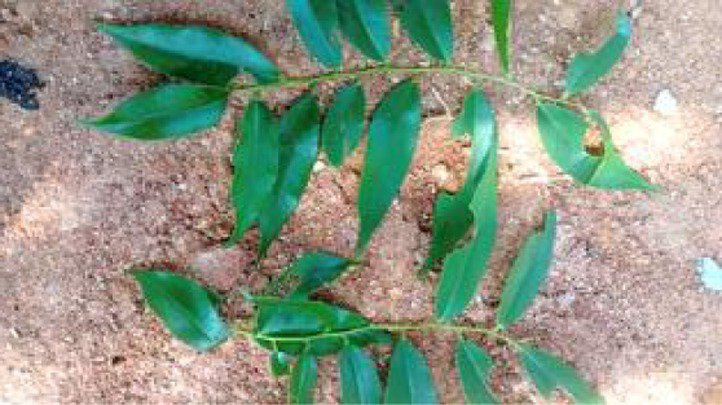 Garo name: *Adurak* Common name: Rohitaka Scientific name: *Antidesmaacidum* Family: Phyllanthaceae

For the purpose of this study, green leafy vegetables (GLVs) were categorized based on their primary source of occurrence and management. Cultivated GLVs refer to species intentionally grown in home gardens or agricultural plots under managed conditions. Jhum field GLVs refer to species found within shifting cultivation systems (locally known as jhum), including both intentionally retained plants and naturally regenerating species within cultivated fields. Jhum is a traditional form of rotational agriculture practiced in Northeast India, in which a patch of land is temporarily cleared through slashing and controlled burning, cultivated for a short period, and subsequently left fallow to regenerate before being reused (11). Forest (foraged) GLVs refer to species collected from uncultivated areas such as forests and fallow lands without deliberate cultivation. It is recognized that these categories are not always mutually exclusive, as certain species may occur across multiple ecological niches or be opportunistically managed within farming systems.

### 3.2 Taxonomic diversity

The 20 identified GLVs belonged to 13 plant families. The most represented families were Lamiaceae and Polygonaceae, followed by Phyllanthaceae. Scientific names were confirmed through consultation with botanists and nutrition researchers.

Two species remained unidentified due to insufficient taxonomic references, highlighting the need for further botanical documentation. Table 1 presents the detailed taxonomic classification of the identified GLVs, highlighting family-wise distribution and confirming species diversity within the study area. The dominance of families such as Lamiaceae and Polygonaceae reflects their ecological adaptability and cultural relevance.

### 3.3 Distribution and conservation status of GLVs

The four-cell analysis revealed considerable variation in the distribution of the identified vegetables.

Several species were widely cultivated by many households and therefore considered relatively secure. These included species such as *Bauhinia acuminata*, *Persicaria chinensis*, and *Paederia foetida*.

In contrast, some species were cultivated only by a small number of households or in limited areas, indicating potential risk of genetic erosion. These species require targeted conservation efforts and promotion within local agricultural systems. Tables 2, 3 shows the distribution and conservation status of GLVs based on four-cell analysis, indicating that while some species are widely cultivated and secure, others are restricted to limited households and areas, suggesting potential vulnerability and need for conservation.

**Table 2:** Four-cell analysis of Darechikgre village, West Garo Hills.

Forms of cultivation	Crops	Key insights	Recommendations
Large area, many households	*Ta·bolchubijak* (Tapioca leaves). *Me·gong* (Dwarf white orchid leaves). *Me·bitchi* (Elliptic rhynochotecum). *Balmatchi* (Anamu). *Samkatchi* (Katuk). *Me·kridunok* (Creeping smartweed). *Donggam* (East India glory bower leaves). *Pasim* (skunk vine leaves). *Agunjareng* (Blue fountain bush).	These crops are secured with high cultural and dietary importance.	Promote value-added products to scale up their economic potential.
Large area, few households	*Adurak* (Rohitaka leaves). *Dadarimet*.	Low adoption despite large cultivation areas; risk of declining interest.	Conduct awareness and training programs to encourage participation.
Small area, many households	*Denggasak* (Amaranth leaves). *Me·kemkem* (East Himalayan begonia leaves).	Crops are culturally significant but limited by land availability.	Integrate into home gardens or intercropping systems.
Small area, few households	*Mejak* (Bitter dock). *Tamilang* (Sweet potato leaves).	Endangered crops facing extinction risk due to low cultivation interest.	Introduce conservation programs (seed banks, farmer cooperatives).

**Table 3:** Four-cell analysis of Daribokgre village, East Garo Hills.

Forms of cultivation	Crops	Key insights	Recommendations
Large area, many households	*Matchaduri* (Fish mint). *Me·bitchi* (Elliptic rhynochotecum). *Donggam* (East India glory bower). *Pasim* (Skunk vine). *Balmatchi* (Anamu). *Agunjareng* (Blue fountain bush). *Me·kridunok* (Creeping smartweed). *Samdambong* (Buckwheat leaves).	These crops are widely cultivated and play a key role in local food systems.	Maintain current cultivation; promote for wider markets.
Large area, few households	*Samjalik* (False mellow). *Medongde* (Bridal’s veil).	Limited household involvement puts these crops at risk despite large areas.	Conduct participatory field schools to boost adoption.
Small area, many households	*Me·gong* (Dwarf white orchid leaves). *Mejak* (Bitter dock). *Ta·milang* (Sweet potato leaves).	Traditional crops with strong household interest but land constraints.	Encourage mixed cropping practices for better land optimization.
Small area, few households	*Dadarimet*. *Derikep*.	Crops are highly at risk of loss in this community.	Urgent interventions: reintroduction via seed banks and ITK documentation.

The four-cell analysis revealed considerable variation in the distribution and management of the identified vegetables. Several species were cultivated across larger areas and by a greater number of households, indicating wider adoption and a higher likelihood of continued cultivation within the community. These included species such as *Bauhinia acuminata*, *Persicaria chinensis*, and *Paederia foetida*.

In contrast, some species were cultivated by relatively few households and/or in limited areas, suggesting restricted distribution and a greater risk of decline in use and potential genetic erosion. Such species may require targeted conservation efforts and promotion within local agricultural systems to ensure their continued availability.

Table 3 presents the distribution of GLVs based on four-cell analysis, illustrating differences in the extent of cultivation and household-level adoption. Species with broader distribution are more likely to be maintained within the farming system, whereas those with limited distribution may be more vulnerable and require focused conservation attention.

### 3.4 Indigenous traditional knowledge

The documentation of indigenous traditional knowledge, as evidenced from Table 4, revealed rich cultural practices associated with the use of GLVs.

**Table 4:** Documentation of ITK of the 20 NUGLVs.

Garo name	Scientific name	Common name	Part of the plants which are consumed traditionally	Medicinal uses (orally or external application)	Traditional culinary uses
*Me·bitchi*	*Rhynchotechumellipticum*	Elliptic Rhynocotecum	Leaves	Utilized for headache relief and wound healing by external application. Served as a vital survival resource during famine periods as capable energy source. Crushed leaves are wrapped in banana leaves and put into hot ash for a few minutes. It is then removed and applied warm to areas of ligament tear and fractured joints. Used as *Mibang*.	Boiled with lentil dal and cooked with fish. Also pickled for preservation.
*Me·gongbijak*	*Bauhinia acuminata*	Dwarf White Orchid Tree/butterfly tree	Leaves, Flowers	No particular medicinal use for the leaves; Roots are used for medicinal purpose	The leaves are boiled or fried with other vegetables. They are used in traditional dishes like *pura* or *kapa*, often combined with dry fish. The leaves are boiled with lentils to enhance flavor and nutrition. They can be wrapped in banana leaves and cooked in hot ash or charcoal.
*Donggam*	*Clorendendrum Indicum*	East Indian glory bower	Leaves	Boiled leaves are consumed to control high blood pressure. The juice extracted from the roots by crushing is consumed to treat hemorrhoids and piles.	The leaves are traditionally used in the preparation of local dishes like pura and kapa. The leaves are cooked alongside lentils, enhancing both nutrition and flavor. The leaves are consumed as fritters (deep-fried) or boiled, showcasing their versatility in cooking.
*Derimet/Dadarimit*	–	–	Leaves, Fruits	The vines of the plant are cut and crushed to extract the juice. The juice is then applied to the wound to stop bleeding. Only few drops of the crushed extract are used for woman by consuming to treat postpartum illness like anemia (*Andime*).	Cooked with banana flowers, crabs, or fish, enhancing the dish’s flavor (no soda needed as it tastes sour).
*Pasim*	*Paederiafoetida*	Skunk Vine/Stink Vine	Leaves, Tubers	Boiled leaves used to treat loose motion. The crushed extract is mixed with water and consume to treat anemia and dysentery.	The leaf is important for cooking pura and kapa. It is also used in dishes that include dry fish.
*Balmatchi*	*Petiveriaalliacea*	Anamu	Leaves, Flowers	No particular medicinal use; Medicinal use only for chickens (not for humans)	The leaves are boiled and used to cook pura. They are also used to prepare kapa with any type of meat, often cooked by wrapping inside banana leaves.
*Agunjareng/Anginjaleng*	*Rotheca serrata*	Blue fountain bush	Leaves, Flowers	No particular medicinal use	Cooked with bamboo shoot. Used to prepare kapa with dry fish.
*Me·kridunok/Me·kridonok*	*Persicaria chinensis*	Chinese knotweed	Leaves	For skin ailment (likely eczema) leaves are put over burning charcoal and the warm leaves are put over the affected skin. For post leech bite, the bleeding spot is treated externally by applying the crushed leaves on the affected area.	Cooked with dry fish, fresh fish, crabs or banana flower (no need to add soda)
*Mejak*	*Rumex obtusifolius*	Bitter Dock	Leaves, Flowers	Oral consumption is avoided during body pain. Crushed leaves are wrapped in banana leaves and put into hot ash for a few minutes to apply in ligament tear and fractured joints. To treat postpartum illness like anemia by applying the crushed leaves on head (*Puronkaa*).	Used to prepare pura and kapa. Boiled or steamed with other vegetables. Sundried and preserved for making pura. Wrapped in banana leaves until it turns yellow to enhance the taste while preparing pura.
*Ta·milangbijak*	*Ipomoea batatas*	Sweet potato leaves	Leaves, tuber	To treat UTI by putting the crushed leaves on head (*Puronkaa*). No consumption if they practice *puronkaa*.	Used to prepare kapa and pura. Fried with other vegetables.
*Matchaduri*	*Houttuynia cordata*	Fish Mint Leaves/Chameleon Plant	Leaves and Roots	Crushed leaves were applied to the head and secured with a cloth (*puronkaa*) to alleviate headaches. For stomach aches, they consumed few fresh leaves to cool the gut. However, it is cautioned against using this remedy during UTIs or *Samicheng*.	Used in chutney and salad. Cooked by wrapping in banana leaves with fresh fish or dry fish.
*Sam·jalik*	*Malvastrumtricuspidatum*	False mellow	Leaves	Boiled leaves are consumed to treat stomach ache and menstrual pain	Leaves are boiled and cooked with soda, dry fish can be added
*Me·dongde*	*Clerodendrum wallichii*	Wallich’s glory bower	Leaves	No particular medicinal use	Used in cooking kapa.
*Samdambong*	*Fagopyrum esculentum*	Buckwheat leaves	Leaves	Used to treat hypertension by consuming the boiled leaves	Cooked with meat, crab, fish (*na.tok*), no need to add soda as it is sour in taste
*Derikep*	–	–	Leaves	Crushed leaves extract are applied to treat swollen body	Cooked with meat and dry fish, no need to add soda as tastes sour
*Ta·bolchubijak*	*Manihot esculenta*	Tapioca leaves	Leaves, Tuber	*Puronkaa* is practiced for man to treat headache and for woman to treat postpartum illness (*Andime*)	Consumed by wrapping inside the banana leaves and baked inside burning charcoal with or without dry fish
*Samkatchi*	*Sauropusandrogynus*	Sweet leaf	Leaves	No particular medicinal use	Cooked or steamed by wrapping inside banana leaves. Used to prepare pura.
*Me.kemkem*	*Begonia roxburghii*	East Himalayan Begonia	Leaves	Traditionally used to treat dysentery by consuming the juice of leaves. The washed and cleaned leaves are put into hollow bamboo and crushed and purified. The juice is then extracted and administered to the person. Orally consumed to treat postpartum bleeding.	Cooked with crabs or fish, soda not used for cooking.
*Denggasak*	*Amaranthus viridis*	Amaranth leaves	Leaves	Crushed leaves are wrapped in banana leaves and put into hot ash and the warm leaves are applied in ligament tear and fractured joints	Sautéed with vegetables. Consumed by boiling the leaves. Used to prepare pura and kapa. Steamed over cooking rice.
*Adurak*	*Antidesmaacidum*	Rohitaka	Leaves	No particular medicinal uses	Cooked with fresh fish and lady’s finger, no soda needed as it tastes sour

Traditional dishes such as *kapa* and *pura* commonly incorporate leafy vegetables along with meat, fish, or rice flour. A unique ingredient used in cooking is *kharchi*, a natural alkaline substance prepared from burnt plantain stems or bamboo.

Several GLVs were also used in traditional medicinal practices. Leaves were commonly boiled, crushed, or heated in ash and applied externally to treat ailments such as headaches, wounds, and sprains. Some plants were also consumed to treat digestive disorders and postpartum conditions.

The practice known as *mibang* involves the consumption of freshly foraged edible plants during forest activities to satisfy hunger and provide energy.

The practice locally referred to as *mibang*, which involves the consumption of selected green leafy vegetables during periods of hunger while working in jhum (shifting cultivation) fields, represents a context-specific subsistence behavior embedded within shifting cultivation systems. Unlike generalized foraging, *mibang* occurs within cultivated or semi-managed landscapes, where workers rely on readily available edible greens to alleviate immediate hunger during field activities. Similar forms of opportunistic consumption of uncultivated or semi-wild edible plants within agricultural settings have been documented across smallholder systems, where “weeds” and volunteer species contribute to daily dietary intake (6, 12). From a subsistence ecology perspective, such practices align with optimal foraging principles, wherein immediate consumption during labor reduces energy deficits and enhances work efficiency (13, 14). In this context, *mibang* may be understood as an adaptive strategy that supports both dietary supplementation and labor sustenance within jhum based livelihoods.

The practice of *mibang*, involving the consumption of selected green leafy vegetables during periods of hunger while working in jhum fields, illustrates a micro-level food security mechanism embedded within Indigenous agroecological systems. Rather than representing incidental or opportunistic eating, *mibang* functions as a real-time dietary buffering strategy that mitigates transient energy deficits during labor-intensive agricultural activities. Such practices highlight the role of uncultivated and semi-wild edible species within managed landscapes as nutritionally significant, yet often overlooked, components of daily diets. Similar contributions of “edible weeds” and volunteer species to dietary diversity and micronutrient intake have been documented in smallholder systems globally (6, 12). From a subsistence ecology perspective, *mibang* aligns with optimal foraging principles, wherein immediate consumption during resource use reduces energetic costs and enhances labor efficiency (13, 14). Importantly, this practice challenges conventional distinctions between cultivated and wild food systems, revealing a continuum in which food access, ecological knowledge, and labor are tightly integrated. Recognizing such practices is critical not only for understanding Indigenous food systems in their full complexity but also for informing context-sensitive strategies aimed at enhancing dietary resilience and nutrition security in marginal environments.

### 3.5 Nutrient composition

Table 5 presents the nutrient and phytoconstituent composition of the selected GLVs, revealing statistically significant variation (*p* < 0.05) across all parameters.

**Table 5:** Analysis of variance (ANOVA) for nutrient composition of the ten selected green leafy vegetables.

Nutrient	SEM	*F* value	Significance (p)	df
Moisture	0.43	471.35	<0.05	9
Ash	0.22	559.74	<0.05	9
Energy	4.89	36.67	<0.05	9
Carbohydrate	1.31	65.26	<0.05	9
Crude fat	0.38	20.39	<0.05	9
Crude protein	0.92	38.78	<0.05	9
Crude fiber	1.14	51.63	<0.05	9
Calcium	6.41	1736.52	<0.05	9
Iron	1.56	436.71	<0.05	9
Zinc	0.63	17.18	<0.05	9
Total phenolic content	5.03	12527.70	<0.05	9
Total flavonoid content	4.36	2200.91	<0.05	9
Vitamin C	5.05	6771.03	<0.05	9

SEM, standard error of mean; df, degrees of freedom.

The analysis of variance (ANOVA) revealed statistically significant differences (*p* < 0.05) in all fourteen analyzed nutritional parameters among the ten green leafy vegetables studied. This indicates substantial variability in their nutrient composition and confirms that each species possesses a distinct nutritional profile. Notably, minerals such as calcium and iron exhibited particularly high *F*-values, suggesting pronounced differences in micronutrient concentrations among the species. Similarly, the high *F*-values observed for total phenolic content, total flavonoid content, and vitamin C indicate considerable variation in antioxidant compounds across the studied vegetables. These findings highlight the nutritional diversity of neglected and underutilized green leafy vegetables and underscore their potential contribution to dietary diversification, micronutrient intake, and overall nutrition security in indigenous food systems.

### 3.6 Comparative nutrient profile of selected GLVs

**Figure 2:**
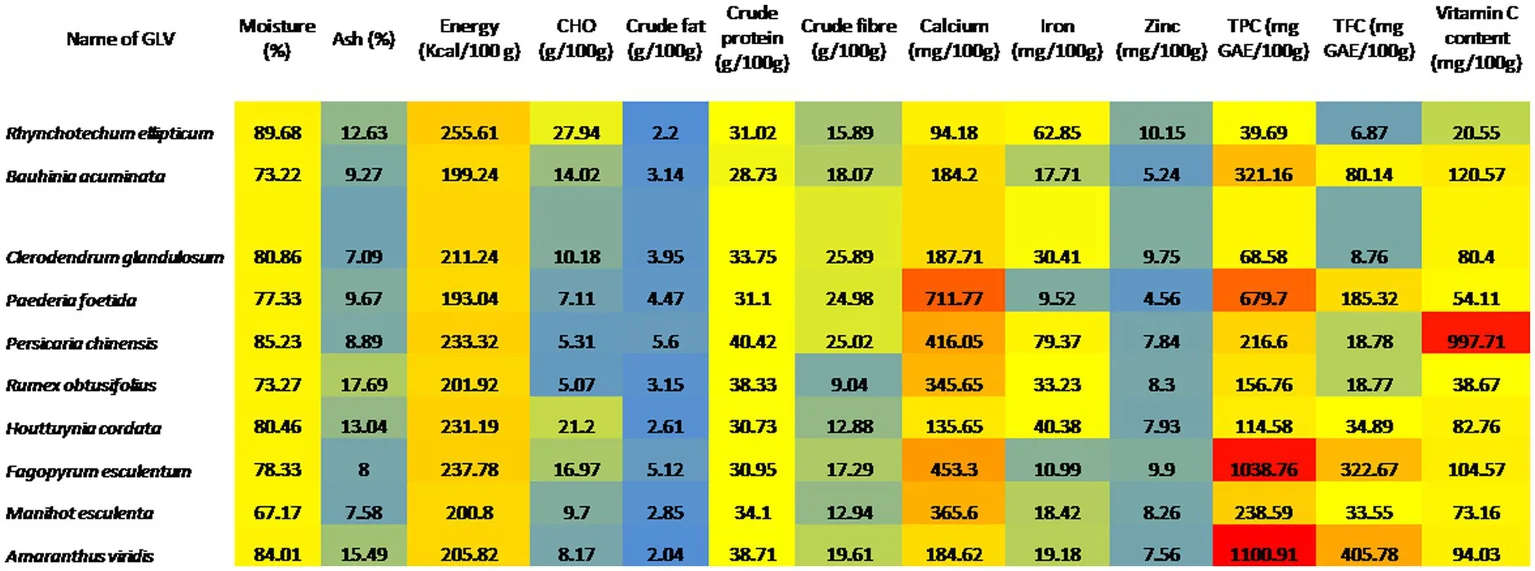
Heatmap illustrating variation in nutrient composition among the ten selected green leafy vegetables.

A heatmap illustrating the comparative nutrient composition of the ten selected green leafy vegetables is presented in Figure 2. The visualization highlights substantial variation in macronutrients, minerals, and antioxidant compounds among species. Certain vegetables exhibited higher concentrations of minerals such as calcium and iron, while others showed elevated levels of phenolic and flavonoid compounds. This variation emphasizes the nutritional diversity of neglected and underutilized leafy vegetables and their potential contribution to dietary diversification and micronutrient security.

## 4 Discussion

The present study highlights the nutritional and cultural significance of neglected and underutilized green leafy vegetables (GLVs) within the indigenous food systems of the Garo Hills. The integration of participatory approaches with laboratory-based nutritional evaluation provided a comprehensive understanding of the role these vegetables play in local diets, traditional knowledge systems, and agrobiodiversity conservation.

### 4.1 Nutritional importance of indigenous leafy vegetables

The proximate analysis conducted in the present study revealed that several of the investigated green leafy vegetables (GLVs) contain substantial amounts of protein, dietary fiber, and essential minerals (Table 6). The analysis of variance further indicated statistically significant differences (*p* < 0.05) among the studied species for all proximate and mineral parameters (Table 5), demonstrating the considerable variability in nutrient composition among the ten GLVs analyzed. Leafy vegetables are widely recognized as inexpensive and accessible sources of micronutrients that are essential for human health, particularly in rural and resource-constrained communities where access to diverse foods may be limited. Studies across Asia and Africa have demonstrated that traditional leafy vegetables contribute significantly to dietary diversity and micronutrient intake, especially iron, calcium, and vitamin C (15, 16). In addition, wild edible plants are increasingly recognized as important contributors to dietary diversity and food security in indigenous communities (12).

**Table 6:** Homogeneity of nutrient content based on Duncan Multiple Range Test (DMRT).

Name of the GLVs	Moisture (%)	Ash (%)	Energy (Kcal/100 g)	CHO (g/100 g)	Crude fat (g/100 g)	Crude protein (g/100 g)	Crude fiber (g/100 g)	Calcium (mg/100 g)	Iron (mg/100 g)	Zinc (mg/100 g)	TPC (mg GAE/100 g)	TFC (mg QE/100 g)	Vitamin C content (mg/100 g)
*Rhynchotechumellipticum*	89.68^h^	12.63^e^	255.61^e^	27.94^h^	2.20^a^	31.02^b^	15.89^c^	94.18^a^	62.85^a^	10.15^c^	39.69^a^	6.87^a^	20.55^a^
*Bauhinia acuminata*	73.22^b^	9.27^cd^	199.24^ab^	14.02^e^	3.14^bc^	28.73^a^	18.07^cd^	184.20^c^	17.71^b^	5.24^a^	321.16^g^	80.14^d^	120.57^g^
*Clerodendrum glandulosum*	80.86^e^	7.09^a^	211.24^c^	10.18^d^	3.95^cd^	33.75^c^	25.89^e^	187.71^c^	30.41^c^	9.75^c^	68.58^b^	8.76^a^	80.40^d^
*Paederiafoetida*	77.33^c^	9.67^d^	193.04^a^	7.11^abc^	4.47^de^	31.10^b^	24.98^e^	711.77^h^	9.52^d^	4.56^a^	679.70^h^	185.32^e^	54.11^c^
*Persicaria chinensis*	85.23^g^	8.89^c^	233.32^d^	5.31^ab^	5.60^f^	40.42^e^	25.02^e^	416.05^f^	79.37^e^	7.84^b^	216.60^e^	18.78^b^	997.71^h^
*Rumex obtusifolius*	73.27^b^	17.69^g^	201.92^abc^	5.07^a^	3.15^bc^	38.33^d^	9.04^a^	345.65^d^	33.23^c^	8.30^b^	156.76^d^	18.77^b^	38.67^b^
*Houttuynia cordata*	80.46^e^	13.04^e^	231.19^d^	21.2^g^	2.61^ab^	30.73^b^	12.88^b^	135.65^b^	40.38^f^	7.93^b^	114.58^c^	34.89^c^	82.76^d^
*Fagopyrum esculentum*	78.33^d^	8.00^b^	237.78^d^	16.97^f^	5.12^ef^	30.95^b^	17.29^cd^	453.30^g^	10.99^d^	9.90^c^	1038.76^i^	322.67^f^	104.57^f^
*Manihot esculenta*	67.17^a^	7.58^b^	200.80^abc^	9.70^cd^	2.85^ab^	34.10^c^	12.94^b^	365.60^e^	18.42^b^	8.26^b^	238.59^f^	33.55^c^	73.16^d^
*Amaranthus viridis*	84.01^f^	15.49^f^	205.82^bc^	8.17^bcd^	2.04^a^	38.71^de^	19.61^d^	184.62^c^	19.18^b^	7.56^b^	1100.91^j^	405.78^g^	94.03e

Superscripts of letters a, b, c denotes statistically significant differences, while superscripts of the same letter denote values that are statistically not significant. Non-overlapping letter indicates significance difference, overlapping letter indicates non-significance difference.

Among the studied species, relatively high protein contents were observed in *Persicaria chinensis* (40.42 g/100 g), *Amaranthus viridis* (38.71 g/100 g), and *Rumex obtusifolius* (38.33 g/100 g). These findings indicate that traditional leafy vegetables can contribute meaningfully to daily protein intake when consumed regularly as part of mixed diets. Although leafy vegetables are not typically considered primary protein sources, their frequent inclusion in traditional meals allows them to make cumulative contributions to dietary protein intake.

The mineral composition of the studied vegetables is particularly noteworthy. High calcium concentrations were recorded in *Paederia foetida* (711.77 mg/100 g), *Fagopyrum esculentum* (453.30 mg/100 g), and *Persicaria chinensis* (416.05 mg/100 g), suggesting that these vegetables may serve as valuable dietary sources of calcium. Calcium plays a crucial role in maintaining bone health and supporting physiological processes such as muscle contraction and nerve function. Previous studies have similarly reported high mineral content in buckwheat leaves, highlighting their potential as nutrient-rich foods (17).

Iron concentrations were also substantial in several of the studied species. Notably, *Persicaria chinensis* exhibited the highest iron content (79.37 mg/100 g), followed by *Rhynchotechum ellipticum* (62.85 mg/100 g). Iron deficiency remains one of the most widespread nutritional problems globally and is a leading cause of anemia, particularly among women and children in developing regions. The presence of considerable iron concentrations in these vegetables therefore indicates their potential role in improving iron intake within traditional diets. Indigenous vegetables have often been reported to contain higher micronutrient concentrations than commonly cultivated vegetables, thereby offering valuable nutritional benefits for rural populations (2, 18).

The calcium content observed in *Paederia foetida* (711.77 mg/100 g) is higher than values reported for several commonly consumed leafy vegetables in previous studies (16), indicating its potential as a superior plant-based calcium source. Similarly, the iron content recorded in *Persicaria chinensis* (79.37 mg/100 g) exceeds values reported for many traditional leafy vegetables in sub-Saharan Africa (15), suggesting its relevance in addressing iron deficiency. These comparisons highlight that selected indigenous GLVs from the Garo Hills are not only nutritionally comparable but, in some cases, nutritionally superior to widely documented traditional vegetables.

Although several of the documented GLVs exhibited substantial levels of calcium, iron, and other micronutrients, the bioavailability of these nutrients may be influenced by the presence of antinutritional compounds such as oxalates, phytates, tannins, and saponins, which are known to affect mineral absorption and nutrient utilization. The present study did not assess these compounds, and therefore the nutritional contribution of the vegetables should be interpreted within this limitation. Future studies should further investigate antinutritional factors, nutrient bioavailability, and the effects of traditional cooking and processing methods on nutrient retention.

Dietary fiber content among the studied GLVs also varied considerably. Species such as *Clerodendrum glandulosum* (25.89 g/100 g) and *Persicaria chinensis* (25.02 g/100 g) showed relatively high fiber levels, which may contribute to improved digestive health and reduced risk of chronic diseases such as cardiovascular disorders and type 2 diabetes.

The heatmap visualization (Figure 2) further illustrates the variation in nutrient density among the studied species, highlighting the complementary nutritional roles that different leafy vegetables may play within traditional diets. Such diversity underscores the importance of consuming a variety of indigenous vegetables to ensure balanced nutrient intake.

Overall, the results of this study demonstrate that neglected and underutilized green leafy vegetables possess considerable nutritional potential and can contribute significantly to dietary diversity and micronutrient security. Promoting their cultivation and consumption within indigenous food systems may therefore represent an effective strategy for addressing micronutrient deficiencies and strengthening sustainable local food systems. These findings align with existing literature while also demonstrating higher micronutrient densities in selected species, reinforcing their relevance in nutrition-sensitive interventions.

### 4.2 Antioxidant potential and health implications

In addition to their macronutrient and mineral composition, the analyzed green leafy vegetables (GLVs) exhibited considerable concentrations of bioactive compounds, including total phenolic content (TPC), total flavonoid content (TFC), and vitamin C. These phytochemicals are widely recognized for their antioxidant properties and their ability to neutralize reactive oxygen species (ROS), thereby protecting cellular components from oxidative damage. The analysis of variance (Table 5) indicated statistically significant differences (*p* < 0.05) among the studied GLVs for all antioxidant-related parameters, including total phenolics, flavonoids, and vitamin C, suggesting substantial variability in their antioxidant potential.

Phenolic compounds exert antioxidant activity by donating hydrogen atoms or electrons to neutralize reactive oxygen species, thereby reducing oxidative stress at the cellular level. Flavonoids further contribute through anti-inflammatory pathways, modulating key enzymes and signaling mechanisms associated with chronic disease progression. These combined effects are particularly relevant in preventing non-communicable diseases such as diabetes and hypertension, which are increasingly prevalent in transitioning rural populations.

Among the analyzed species, *Amaranthus viridis* recorded the highest total phenolic content (1100.91 mg GAE/100 g) and flavonoid content (405.78 mg QE/100 g), followed by *Fagopyrum esculentum*, which showed high phenolic (1038.76 mg GAE/100 g) and flavonoid levels (322.67 mg QE/100 g). These values indicate that these species may serve as particularly rich sources of dietary antioxidants. Similar observations have been reported in previous studies of traditional leafy vegetables, where high phenolic concentrations were associated with strong antioxidant activity and potential health benefits (19). Phenolic compounds are known to exhibit anti-inflammatory, antimicrobial, and cardioprotective properties, thereby contributing to the prevention of several chronic diseases.

Flavonoids, another important group of plant secondary metabolites, were also present in substantial concentrations in several of the studied species. The high flavonoid levels observed in *Amaranthus viridis* and *Fagopyrum esculentum* are consistent with earlier reports indicating that buckwheat leaves and traditional leafy vegetables often contain elevated flavonoid concentrations (20). These compounds play an important role in scavenging free radicals and protecting biological tissues from oxidative stress.

Vitamin C content among the analyzed GLVs also showed considerable variation. Notably, *Persicaria chinensis* exhibited the highest vitamin C content (997.71 mg/100 g), followed by *Bauhinia acuminata* (120.57 mg/100 g) and *Fagopyrum esculentum* (104.57 mg/100 g). Vitamin C is a potent antioxidant that supports immune function, collagen synthesis, and cellular protection against oxidative damage. The co-occurrence of vitamin C and iron in several GLVs such as *Persicaria chinensis* enhances non-heme iron absorption, thereby improving iron bioavailability and strengthening their role in combating iron deficiency anemia.

The simultaneous presence of iron and vitamin C in several of the studied species may therefore provide important synergistic nutritional benefits. For instance, *Persicaria chinensis* not only exhibited high vitamin C levels but also contained substantial iron concentrations (79.37 mg/100 g), suggesting its potential role in improving iron nutrition when incorporated into traditional diets.

The heatmap visualization (Figure 2) further illustrates the variation in antioxidant compounds among the studied GLVs, highlighting species-specific nutrient patterns and reinforcing the complementary roles that different vegetables may play within traditional diets. Such diversity in antioxidant compounds underscores the potential health benefits of consuming a variety of traditional leafy vegetables.

Overall, the findings of this study demonstrate that neglected and underutilized green leafy vegetables possess significant antioxidant potential and may contribute to protective health effects when incorporated into regular diets. Promoting their consumption may therefore support both improved micronutrient intake and enhanced antioxidant protection, particularly in rural and indigenous communities.

### 4.3 Indigenous knowledge and cultural significance

The study documented a rich body of indigenous traditional knowledge associated with the identification, preparation, and medicinal use of neglected and underutilized green leafy vegetables (GLVs) among the Garo communities of Meghalaya. These vegetables are deeply embedded in the local food culture and form an integral part of everyday diets as well as seasonal foraging practices. Traditional dishes such as *kapa* and *pura* commonly incorporate leafy vegetables together with meat, fish, or rice flour and represent important components of the Garo culinary tradition. These dishes are not only culturally significant but also nutritionally balanced, combining plant-based micronutrients with animal protein sources.

A distinctive feature of traditional Garo cooking is the use of *kharchi*, a natural alkaline substance obtained from the ash of burnt plantain stems or bamboo. This ingredient is traditionally added during cooking to enhance flavor, soften fibrous plant tissues, and improve digestibility of leafy vegetables. Similar alkaline processing techniques have been reported in other indigenous cuisines of Northeast India and Southeast Asia, where plant ash is used to modify taste and texture while also influencing nutrient bioavailability.

The indigenous knowledge recorded in this study also highlights the medicinal importance of several GLVs. Community members reported using specific species to treat common ailments such as headaches, digestive disorders, wounds, hypertension, and postpartum conditions. For example, leaves of *Rhynchotechum ellipticum* and *Rumex obtusifolius* are traditionally heated in ash and applied externally to treat ligament injuries or fractures, while *Paederia foetida* leaves are consumed to alleviate digestive disturbances. Similarly, *Begonia roxburghii* is traditionally used for treating dysentery and postpartum bleeding. These ethnomedicinal practices demonstrate how food plants serve dual roles as both dietary components and therapeutic resources within indigenous healthcare systems. Such food-medicine continuums are widely recognized in ethnobotanical research and reflect the holistic understanding of health maintained by many traditional societies (21).

Another culturally significant practice documented in the study is *mibang*, which involves the consumption of freshly foraged edible plants during forest activities or agricultural work. This practice reflects the intimate ecological knowledge possessed by community members regarding the identification of edible species in their natural environment. Mibang not only provides an immediate source of nourishment during long working hours but also reinforces the cultural relationship between people and surrounding ecosystems.

Indigenous leafy vegetables therefore represent more than simply food resources; they are closely linked to cultural identity, ecological knowledge, and traditional healthcare practices. Similar patterns have been observed in indigenous communities worldwide, where wild and semi-domesticated vegetables contribute significantly to dietary diversity, resilience of local food systems, and preservation of cultural heritage (6, 7). However, the erosion of traditional knowledge due to changing food habits, urbanization, and generational shifts poses a risk to the continued use of these valuable plant resources.

Preserving and promoting indigenous knowledge related to traditional vegetables is therefore essential not only for safeguarding cultural heritage but also for supporting sustainable and nutrition-sensitive food systems. Documentation of such knowledge, as undertaken in this study, can contribute to the recognition of indigenous food resources and encourage their integration into contemporary nutrition and agricultural development initiatives. The nutritional richness observed in several of the studied species further supports the empirical knowledge of local communities who have long recognized these plants as valuable dietary resources.

### 4.4 Conservation and food system implications

The four-cell analysis provided important insights into the conservation status of the identified GLVs. While some species were widely cultivated and commonly consumed, others were restricted to small areas or cultivated by only a few households. Such patterns indicate potential risks of genetic erosion and loss of traditional food resources.

The declining consumption of traditional leafy vegetables has been reported in many parts of the world due to dietary transitions, urbanization, and the increasing availability of commercial foods. Promoting awareness of the nutritional value of indigenous vegetables and integrating them into local food systems could help address this decline.

The findings of this study highlight the importance of integrating traditional knowledge with scientific research to support the conservation and promotion of neglected food plants. Encouraging their cultivation in home gardens and community farms, along with value addition and market development, could contribute to sustainable food systems and improved nutrition. Conservation and promotion of neglected crops are essential for maintaining agricultural biodiversity and improving sustainable food systems (4, 5).

## 5 Study limitations

Despite the valuable insights generated by this study, several limitations should be acknowledged. First, the study was conducted in only two villages within the Garo Hills region, which may limit the generalizability of the findings to other communities in Meghalaya or northeastern India. Indigenous food systems are often highly localized, and variations in plant use and traditional knowledge may exist across different geographical and cultural contexts. The voucher specimens were not deposited for herbarium authentication, as the work primarily focused on nutritional profiling rather than taxonomic revision; although species identification was conducted in consultation with experts from the North East Society for Agroecology Support, supported by local knowledge custodians and cross-verified using standard regional floras, future studies should incorporate formal voucher deposition to strengthen taxonomic validation. In addition, although women emerged as important custodians of culinary and ethnobotanical knowledge during community interactions, a gender-disaggregated analysis of GLV knowledge, use practices, and Indigenous Traditional Knowledge (ITK) transmission was not systematically undertaken in the present study. Future research may further explore gendered dimensions of Indigenous food systems and the role of women in preserving and transmitting traditional ecological knowledge.

Second, nutrient analysis was carried out for only ten of the twenty identified green leafy vegetables due to constraints related to sample availability and laboratory resources. Although these species were selected based on cultural relevance and availability, additional studies involving a larger number of species would provide a more comprehensive understanding of the nutritional potential of indigenous leafy vegetables in the region.

Third, the study focused primarily on proximate composition, selected minerals, vitamin C, and selected phytochemicals. Other important nutritional components, such as carotenoids, folates, and additional micronutrients, were not analyzed and could be explored in future studies. Furthermore, antinutritional factors such as oxalates, phytates, tannins, and saponins, which may influence mineral bioavailability and nutrient utilization, were not assessed. Nutritional analyses were conducted on a dry weight basis, and the effects of traditional cooking and processing methods on nutrient retention were also not evaluated. Future research should therefore investigate nutrient bioavailability, antinutritional compounds, fresh and cooked weight nutrient equivalents, and cooking retention effects to better understand the dietary significance of these vegetables under actual consumption conditions.

Finally, although Indigenous Traditional Knowledge (ITK) regarding the medicinal uses of the documented green leafy vegetables was recorded through community interactions, the pharmacological properties of these plants were not experimentally validated in the present study. Furthermore, it is important to emphasize that such knowledge is collectively held by Indigenous communities, who act as its primary custodians. Any further scientific validation or application of this knowledge must be undertaken with prior informed consent and in accordance with equitable benefit-sharing principles, as outlined in frameworks such as the Convention on Biological Diversity and the Nagoya Protocol (8, 9). In light of ongoing concerns surrounding biopiracy and the misappropriation of traditional knowledge (22, 23), the present study does not assert novelty or proprietary claims over the documented knowledge but seeks to support its recognition within appropriate ethical boundaries.

## 6 Policy and nutrition implications

The findings of this study have important implications for food security, nutrition policy, and agrobiodiversity conservation in northeastern India. The high nutrient density and antioxidant properties observed in several of the studied green leafy vegetables highlight their potential role in addressing micronutrient deficiencies, particularly iron and calcium deficiencies, which remain prevalent in many rural communities.

Promoting the cultivation and consumption of indigenous leafy vegetables could contribute to improving dietary diversity and strengthening nutrition-sensitive agriculture. These vegetables are often well adapted to local environmental conditions, require minimal external inputs, and can be cultivated in home gardens, making them accessible and sustainable food sources for rural households.

The documentation of indigenous knowledge also underscores the importance of preserving traditional food systems and intergenerational knowledge transfer. Community-based initiatives such as nutrition education programs, farmer field schools, and school garden projects could help raise awareness about the nutritional benefits of these traditional vegetables.

At the policy level, integrating neglected and underutilized species into government nutrition and agriculture programs could enhance their visibility and utilization. Initiatives such as the promotion of indigenous vegetables in public food distribution systems, mid-day meal programs, and community nutrition schemes could support both improved nutrition outcomes and biodiversity conservation.

Furthermore, supporting value addition, small-scale processing, and local market development for these vegetables may create new livelihood opportunities for rural communities, particularly women who are often the primary custodians of traditional food knowledge.

## 7 Conclusion

This study provides comprehensive documentation and nutritional evaluation of neglected and underutilized green leafy vegetables (GLVs) traditionally consumed by indigenous communities in the Garo Hills of Meghalaya. Using participatory approaches, twenty GLVs belonging to thirteen plant families were identified, and their associated indigenous traditional knowledge related to culinary practices and medicinal uses was documented. Nutrient profiling of selected species revealed considerable variation in proximate composition, mineral content, and bioactive compounds, with several vegetables exhibiting substantial levels of protein, dietary fiber, calcium, iron, and antioxidant compounds. The analysis of variance confirmed statistically significant differences among the studied species, highlighting their distinct nutritional profiles.

The findings demonstrate that many of these traditional vegetables possess significant nutritional and antioxidant potential and may contribute meaningfully to dietary diversity and micronutrient intake. Species such as *Persicaria chinensis*, *Amaranthus viridis*, and *Paederia foetida* were particularly notable for their high nutrient and bioactive compound content. The presence of both micronutrients and antioxidant compounds in these vegetables underscores their potential role as functional foods capable of supporting improved nutritional and health outcomes.

In addition to their nutritional value, the study highlights the importance of indigenous traditional knowledge in shaping food practices and maintaining agrobiodiversity. Traditional culinary practices, medicinal uses, and foraging behaviors such as *mibang* reflect the close ecological relationship between the Garo people and their surrounding environment. These knowledge systems play a crucial role in sustaining local food systems and preserving culturally important plant resources.

Importantly, many of the documented GLVs are well adapted to marginal environments and thrive under low-input conditions characteristic of jhum systems, indicating their potential role in climate-resilient food systems. Their ability to grow under conditions of ecological variability, including periods of limited water availability and soil fertility, reinforces their relevance in the context of climate change and shifting agricultural landscapes.

Overall, neglected and underutilized leafy vegetables represent valuable yet underrecognized components of indigenous food systems. Promoting their conservation, cultivation, and consumption could contribute to improved nutrition security, dietary diversification, and sustainable food systems in northeastern India. Future research should further explore the bioavailability of nutrients, pharmacological properties of these plants, and opportunities for value addition and community-based promotion of traditional vegetables.

## Data Availability

The original contributions presented in the study are included in the article/supplementary material, further inquiries can be directed to the corresponding author.
